# Improving the use of research evidence in guideline development: 15. Disseminating and implementing guidelines

**DOI:** 10.1186/1478-4505-4-27

**Published:** 2006-12-08

**Authors:** Atle Fretheim, Holger J Schünemann, Andrew D Oxman

**Affiliations:** 1 Norwegian Knowledge Centre for the Health Services, P.O. Box 7004, St. Olavs plass, N-0130 Oslo, Norway; 2 INFORMA, S.C. Epidemiologia, Istitituto Regina Elena, Via Elio Chianesi 53, 00144 Rome, Italy

## Abstract

**Background:**

The World Health Organization (WHO), like many other organisations around the world, has recognised the need to use more rigorous processes to ensure that health care recommendations are informed by the best available research evidence. This is the 15^th ^of a series of 16 reviews that have been prepared as background for advice from the WHO Advisory Committee on Health Research to WHO on how to achieve this.

**Objectives:**

In this review we address strategies for the implementation of recommendations in health care.

**Methods:**

We examined overviews of systematic reviews of interventions to improve health care delivery and health care systems prepared by the Cochrane Effective Practice and Organisation of Care (EPOC) group. We also conducted searches using PubMed and three databases of methodological studies for existing systematic reviews and relevant methodological research. We did not conduct systematic reviews ourselves. Our conclusions are based on the available evidence, consideration of what WHO and other organisations are doing and logical arguments.

**Key questions and answers:**

What should WHO do to disseminate and facilitate the uptake of recommendations?

• WHO should choose strategies to implement their guidelines from among those which have been evaluated positively in the published literature on implementation research

• Because the evidence base is weak and modest to moderate effects, at best, can be anticipated, WHO should promote rigorous evaluations of implementation strategies.

What should be done at headquarters, by regional offices and in countries?

• Adaptation and implementation of WHO guidelines should be done locally, at the national or sub-national level.

• WHO headquarters and regional offices should support the development and evaluation of implementation strategies by local authorities.

## Background

The World Health Organization (WHO), like many other organisations around the world, has recognised the need to use more rigorous processes to ensure that health care recommendations are informed by the best available research evidence. This is the 15th of a series of 16 reviews that have been prepared as background for advice from the WHO Advisory Committee on Health Research to WHO on how to achieve this.

Developing recommendations makes little sense if they are not used. Thus, effective strategies to promote the appropriate use of recommendations by decision-makers (clinicians, public health officers, policymakers) are important.

In this paper we address the following questions:

• What should WHO do to disseminate and facilitate the uptake of recommendations?

• What should be done at headquarters, by regional offices and in countries?

Questions related to adaptation and evaluation of guidelines are addressed in other papers in this series [1,2].

## What is WHO doing now?

There is no general WHO strategy for guideline implementation. The methods that are used vary from department to department, and may vary from case to case within departments. Field testing and rollout strategies that are used to promote the uptake of recommendations are often not informed by the findings of implementation research, and it is generally difficult to estimate the impact of the implementation strategies that are used, since evaluations are rarely rigorous, if they are done at all.

There are, however, examples of rigorous evaluations, such as implementation of the Integrated Management for Childhood Illnesses (IMCI) guideline, which has been evaluated in a randomised controlled trial [3]. WHO has also in some cases reviewed the relevant evidence-base, for example for strategies to improve the use of drugs in developing countries [4].

Although recommendations inevitably need to be adapted and implemented at country level, WHO headquarters and regional offices can support these activities [2].

## What are other organisations doing?

In an international survey of organisations that develop guidelines or health technology assessments, almost half of the 95 respondents reported using provider-mediated interventions as part of their strategy for implementing of guidelines [5]. Examples of this were conducting audits and hosting work-shops for practitioners. However, many respondents did not provide specific examples.

In a smaller international survey of prominent guideline developers, nearly all of the 18 organisations used educational materials and conferences as part of their implementation strategies [6]. Other common approaches were audit and feed-back, use of local opinion leaders, and organizational interventions (e.g. financial incentives or disincentives).

## Methods

The methods used to prepare this review are described in the introduction to this series [7]. The key questions addressed in this paper were vetted amongst the authors and the ACHR Subcommittee on the Use of Research Evidence (SURE). The Cochrane Effective Practice and Organisation of Care (EPOC) group undertakes systematic reviews of interventions to improve health care delivery and health care systems. EPOC has undertaken periodic overviews of systematic reviews to assess and summarise the evidence available from existing Cochrane and non Cochrane reviews [8-10]. The research findings reported here are drawn from these overviews and an update of those overviews that is underway. In addition, we searched PubMed and three databases of methodological literature (within the databases of The Cochrane Library, the US National Guideline Clearinghouse [11] and the Guidelines International Network [12]) for existing systematic reviews and relevant methodological research that address these questions. The search-term we used was "guidelines and implementation and systematic review".

We did not conduct systematic reviews ourselves. The answers to the questions are our conclusions based on the available evidence, consideration of what WHO and other organisations are doing and logical arguments.

## Findings

### What should WHO do to disseminate and facilitate the uptake of recommendations?

Most research on implementation and dissemination strategies for guidelines have focused on clinical practice guidelines, with change in clinical practice being the primary outcome of interest. An overview of systematic reviews of interventions aimed at changing provider behaviour found that: "In general, passive approaches are generally ineffective and unlikely to result in behaviour change. Most other interventions are effective under some circumstances; none are effective under all circumstances. Promising approaches include educational outreach (for prescribing) and reminders" [9]. A more recent comprehensive review of evaluations of the effects of strategies for guideline implementation found that "The majority of interventions observed modest to moderate improvements in care", but there was "considerable variation in the observed effects both within and across interventions"[13].

Few evaluations of interventions to change professional practice have been conducted in low-income countries [14].

Guidance from WHO is often directed towards policymakers. There is limited research to inform the choice of strategies to improve the uptake of WHO recommendations by policymakers. The findings of systematic reviews of studies of decision-making by health care managers and policymakers have found that factors such as interactions between researchers and health care policy-makers and timing/timeliness appear to increase the prospects for research use among policymakers [15,16].

### What should be done at headquarters, by regional offices and in countries?

We did not identify any research findings that could inform the answer to this question.

## Discussion

Passive dissemination of guidelines alone is not likely to adequately ensure appropriate uptake of recommendations in most circumstances. However, the conclusion in an extensive review of guidelines implementation strategies was: "There is an imperfect evidence base to support decisions about which guideline dissemination and implementation strategies are likely to be efficient under different circumstances" [13]. Thus, WHO needs to carefully consider the likely benefits and costs of alternative implementation strategies in relationship to specific contexts, and to evaluate the impact of selected strategies.

There are tools available that are designed to assist in the design and evaluation of implementation strategies, such as NorthStar, developed by the EC-funded Research-based continuing education and quality improvement (ReBEQI) project [17]. NorthStar provides a range of information, checklists, examples and tools based on current research on how to best design and evaluate implementation strategies.

Health authorities at national or sub-national levels are better able than WHO to tailor implementation strategies to their specific circumstances. However, they frequently lack capacity and resources to do this. WHO headquarters and regional offices can play an important role in supporting member states in their efforts to implement recommendations by providing tools such as NorthStar, support and coordination of efforts.

## Further work

Rigorous evaluations of the effectiveness of strategies for implementing and disseminating recommendations are needed. Given that the use and impact of WHO recommendations is likely to be limited without an active implementation strategy, it is of paramount interest to the organisation to invest in generating the knowledge needed for successful implementation.

## Competing interests

AF and ADO work for the Norwegian Knowledge Centre forthe Health Services, an agency funded by the Norwegian government that produces systematic reviews and health technology assessments. All three authors are contributors to the Cochrane Collaboration. ADO and HJS are members of the GRADE Working Group. HJS is documents editor and chair of the documents development and implementation committee for the American Thoracic Society and senior editor of the American College of Chest Physicians' Antithrombotic and Thrombolytic Therapy Guidelines.

## Authors' contributions

AF prepared the first draft of this review. HJS and ADO contributed to drafting and revising it. All authors read and approved the final manuscript.
